# Exploring the Current Use of Educational Technologies in the Oncology Domain Across Europe: A Cross-Sectional Study

**DOI:** 10.1007/s13187-025-02703-1

**Published:** 2025-08-29

**Authors:** Jana Steinbacher, Taibe Kulaksız, Marco Kalz

**Affiliations:** 1 Institut für Bildungsanalysen Baden-Württemberg, Stuttgart, Germany; 2 Heidelberg University of Education, Heidelberg, Germany

**Keywords:** Digital education, Oncology training, Technology-enhanced learning, Multi-disciplinary learning, Educational technology

## Abstract

**Background:**

Considering the increasing relevance of digital education in cancer training, this study explores the current use of educational technologies in oncology across Europe.

**Methods:**

A cross-sectional study was conducted using convenience sampling to gather responses from learners and educators across Europe. Data was collected online via Lime Survey.

**Results:**

Results indicate that both groups are most familiar with synchronous and blended learning, with less experience in asynchronous and hybrid formats. Live lectures were the most used tool, while virtual reality and simulations were less used. Regional differences reveal that Northern Europe prefers audio-based tools and e-books, while Central Europe demonstrates higher familiarity with asynchronous learning and interactive tools. Southern Europe has less experience with various modalities and tools. Professionally, cancer nurses reported more experience with asynchronous learning and learning management systems, while clinical oncologists demonstrated limited familiarity with various tools, particularly emerging technologies.

**Conclusions:**

For multi-disciplinary oncology training to succeed, practitioners need to align learning objectives with participants’ prior experiences and balance diverse target groups and implementation needs to address regional and professional disparities. Targeted efforts are needed to bridge gaps in digital infrastructure, accessibility, and institutional support. Explanatory studies are needed to confirm these findings.

**Supplementary Information:**

The online version contains supplementary material available at https://doi.org/10.1007/s13187-025-02703-1.

## Introduction

One of the key factors in improving cancer care is to enhance the education of oncology healthcare professionals. Technology-enhanced learning (TEL) is increasingly recognised for its role in the training and professional development of healthcare staff, particularly in oncology [1]. TEL offers innovative solutions to meet the growing need for continuous learning in cancer care by providing flexible, accessible, yet effective training.

However, the implementation of innovative educational technologies requires an extensive analysis of previous experiences for innovations to be within a reachable distance [2]. The use of digital teaching methods, especially independent asynchronous learning, can be challenging for students [3]. Besides, healthcare professionals (e.g., in radiation oncology and paediatric oncology) might have varying levels of experience with digital medical education, especially in rural or low-resource settings [4–6].

Europe’s Beating Cancer Plan has initiated numerous projects to improve cancer patient outcomes and patient care across Europe [7]. One of these EU-funded projects, INTERACT-EUROPE, aimed at developing an inter-specialty cancer training programme (ISCTP) involving all main oncology disciplines and professions, cancer centres, and patient groups, based on relevant needs assessments. Its purpose is to foster mutual understanding, communication, and collaboration of different oncological departments, rather than having them work in silos [8]. As part of this project, a curriculum was developed “[to] provide education and training to enable those in specialist training to learn to work more effectively with different specialties and professions […] to deliver better care and provide psychosocial and nutritional support for cancer patients” (ibid., p. 3).

In the INTERACT-EUROPE project, the authors were responsible for developing TEL scenarios for the ISCTP. A scoping review showed that a wide variety of digital tools were employed in cancer training, despite a lack of cutting-edge technologies and only minimal functional improvements compared to conventional training methods [9]. Furthermore, the findings indicated that, while training programmes often yielded favourable results, experimental study designs were comparatively scarce. Still, experimental studies remain a necessity to reveal TEL’s contribution and limitations in cancer education.

As the training programme will be implemented in 100 cancer centres across Europe within the follow-up project INTERACT-EUROPE 100, indications should be found as to whether certain regions or professions require special support. Therefore, to complement our initial study results, we decided to conduct a stakeholder analysis with potential participants and trainers of the ISCTP to develop the most suitable and rich TEL scenarios for our target group. Informed by the concept of “proximal implementation” [2], we believe that we need to gradually bridge from the current situation to the desired state of oncology education for innovations to be prone to successful and sustainable implementation.

## Research Aim

Considering the growing importance of digital education formats and technologies in cancer education, the study aimed to explore the current use of educational technologies in the oncology domain across Europe to determine the degree of innovation that can be accomplished by participants and trainers of the ISCTP. By conducting a cross-sectional survey, we sought to explore how digital education tools are being used in oncology training. Understanding the experiences and challenges faced by cancer healthcare professionals can help guide future efforts to optimise the use of educational technologies in cancer care across Europe. This study addresses a critical gap in the literature by providing an explorative overview of the current state of digital education in oncology, highlighting both opportunities and barriers that exist based on previous experiences. The findings will inform policymakers, educational institutions, and healthcare organisations on how to better support cancer healthcare professionals in their continuing training and professional development through digital means.

## Methods

In this study, a convenience sampling strategy for both learners and educators was selected. The calls for participation were distributed via email among the INTERACT-EUROPE consortium. The surveys were open to participants from EU member states. The different countries have been clustered as follows: Central Europe (Austria, Croatia, Czech Republic, Estonia, Germany, Hungary, Latvia, Lithuania, Luxembourg, Poland, Slovakia, Slovenia), Northern Europe (Denmark, Finland, Sweden), South Eastern Europe (Bulgaria, Republic of Cyprus, Greece, Romania), Southern Europe (Italy, Malta, Portugal, Spain), and Western Europe (Belgium, France, Ireland, Netherlands). Respondents from non-EU countries (“other”) have been excluded from the data analysis. The surveys were conducted online via Lime Survey, i.e., in a computer-assisted self-administered interviewing (CASI) mode. Ethical approval for the study was obtained from the Joint Ethics Committee of Heidelberg University of Education and SRH University Heidelberg. All participants joined the study voluntarily by giving their informed consent.

In this study, the potential participants of the ISCTP were called “learners” and the potential trainers and mentors of the programme were called “educators”. Both learners and educators were asked to rate their previous experiences with different *teaching/learning modalities* (“Please specify how much experience you have with the following technology-enhanced learning scenarios as a learner/student”, “Please specify how much experience you have with the following technology-enhanced learning scenarios as an educator/teacher”) and *tools* (“Please specify how experienced you are with the following learning technologies and formats”). The modalities comprised synchronous learning, asynchronous learning, blended learning, and hybrid learning (with explanations and examples); the tools comprised Learning Management Systems (LMS), mobile learning applications, Massive Open Online Courses (MOOCs), Open Educational Resources (OER), Virtual Reality (VR)/Augmented Reality (AR)/Mixed Reality (XR), digital simulations, live lecture platforms, e-portfolios, e-assessment tools, interactive presentations, video tutorials, podcasts/audio lectures/audiobooks, e-books, online discussion boards/forums/wikis, digital educational games, and social media (with explanations and examples). All items were answered on a 5-point scale (1 = no experience, 2 = some experience, 3 = medium experience, 4 = much experience, 5 = a lot experience). Gender differences were taken into consideration for the design of the study. The gender of participants was defined based on self-report (female, male, non-binary). The overview of the sample is shown in Table 1.

**Table 1:** Sample overview

	Learners	Educators
Sample size	*N* = 112	*N* = 73
Profession		
Cancer Nurse	*N* = 17 (15.2%)	*N* = 8 (11.0%)
Clinical Oncologist	*N* = 4 (3.6%)	*N* = 5 (6.8%)
Medical Oncologist	*N* = 14 (12.5%)	*N* = 14 (19.2%)
Radiation Oncologist	*N* = 20 (17.9%)	*N* = 5 (6.8%)
Surgical Oncologist	*N* = 15 (13.4%)	*N* = 10 (13.7%)
Pathologist	*N* = 17 (15.2%)	*N* = 3 (4.1%)
Region		
Central Europe	*N* = 26 (23.2%)	*N* = 15 (20.5%)
Northern Europe	*N* = 4 (3.6%)	*N* = 7 (9.6%)
South Eastern Europe	*N* = 36 (32.1%)	*N* = 8 (11.0%)
Southern Europe	*N* = 24 (21.4%)	*N* = 28 (38.4%)
Western Europe	*N* = 22 (19.6%)	*N* = 15 (20.5%)
Gender		
Female	*N* = 67 (59.8%)	*N* = 39 (53.4%)
Male	*N* = 45 (40.2%)	*N* = 34 (46.6%)
Age	*M* = 37.49 (*SD* = 8.80)	*M* = 47.18 (*SD* = 11.04)

## Results

### Overall experience with modalities and tools based on target groups

#### Modalities

Both learners and educators are more experienced with synchronous learning (*M* = 3.65; *M* = 3.79) and blended learning (*M* = 3.37; *M* = 3.45), and less experienced with asynchronous learning (*M* = 3.27; *M* = 3.00) and hybrid learning (*M* = 3.29; *M* = 3.35). The learners’ and educators’ experiences with learning modalities in detail are provided in the attachment.

#### Tools

Both learners and educators are most experienced with live lecture platforms (*M* = 3.74; *M* = 4.02). Besides, learners also have much experience with e-books (*M* = 3.60), video tutorials (*M* = 3.51), and podcasts/audio lectures/audiobooks (*M* = 3.51). Learners and educators are less experienced with e.g. e-portfolios (*M* = 2.24; *M* = 2.08), MOOCs (*M* = 2.24; *M* = 2.05), e-assessment tools (*M* = 2.04; *M* = 2.06), VR/AR/XR (*M* = 1.93; *M* = 1.98), and digital simulations (*M* = 2.03; *M* = 1.89). The learners’ and educators’ experiences with digital learning tools in detail are provided in the attachment.

### Experience with Modalities and Tools Based on Regions

#### Modalities

##### Learners

**Figure 1:**
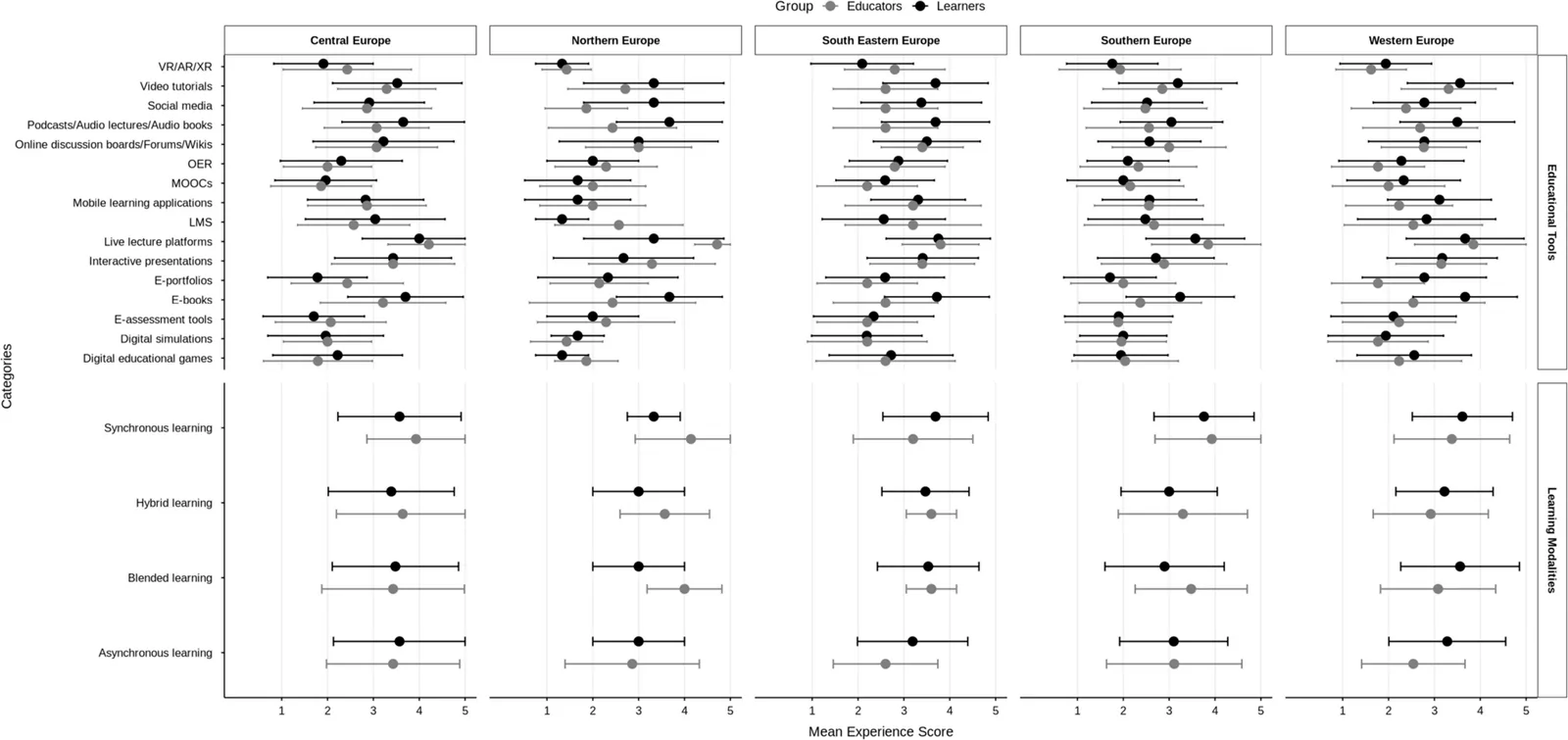
Experience with modalities and tools based on regions

Compared to the overall sample, a similar pattern can be found across regions (Fig. 1). As to synchronous learning, only learners from Northern Europe are less experienced (*M* = 3.33). As to blended learning, learners from Northern Europe (*M* = 3.00) and Southern Europe (*M* = 2.90) have less experience than the overall target group. Learners from Central Europe are more experienced with asynchronous learning (*M* = 3.57) compared to the overall sample. Learners from South Eastern Europe have more experience with hybrid learning (*M* = 3.47) than the overall target group.

##### Educators

Compared to the overall sample, a similar pattern can be found across regions (Fig. 1). As to synchronous learning, educators from South Eastern Europe (*M* = 3.20) and Western Europe (*M* = 3.38) have less experience than the overall target group. As to blended learning, only educators from Western Europe are less experienced (*M* = 3.08). In Western Europe, a low value can also be found with respect to asynchronous learning (*M* = 2.54). Once more, educators from Central Europe have more experience with asynchronous learning (*M* = 3.43) than the overall target group. Educators from Central Europe (*M* = 3.64), Northern Europe (*M* = 3.57), and South Eastern Europe (*M* = 3.60) are more experienced with hybrid learning compared to the overall sample.

#### Tools

##### Learners

Compared to the overall sample, a similar pattern can be found across regions (Fig. 1). Except for learners from Northern Europe (*M* = 3.33), learners are most experienced with live lecture platforms. In Northern Europe, learners are most experienced with podcasts/audio lectures/audiobooks (*M* = 3.67) and e-books (*M* = 3.67). Across regions, learners have much experience with podcasts/audio lectures/audiobooks and e-books, except in Southern Europe (*M* = 3.05; *M* = 3.24), and much experience with video tutorials, except in Northern Europe (*M* = 3.33) and Southern Europe (*M* = 3.19). Besides, learners from Central Europe (*M* = 3.43) and South Eastern Europe (*M* = 3.41) have much experience with interactive presentations. Learners from South Eastern Europe are also well experienced with online discussion boards/forums/wikis (*M* = 3.50). Striking values can be found in the following regions: Learners from Central Europe have no experience with e-portfolios (*M* = 1.78) and e-assessment tools (*M* = 1.70). Learners from Northern Europe have no experience with mobile learning applications (*M* = 1.67), MOOCs (*M* = 1.67), digital simulations (*M* = 1.67), LMS (*M* = 1.33), VR/AR/XR (*M* = 1.33), and digital educational games (*M* = 1.33). Learners from Southern Europe have no experience with VR/AR/XR (*M* = 1.76) and e-portfolios (*M* = 1.71).

##### Educators

Compared to the overall sample, a similar pattern can be found across regions (Fig. 1). Across regions, educators are most experienced with live lecture platforms. Educators from Central Europe (*M* = 4.21) and Northern Europe (*M* = 4.71) indicate the highest values. Once more, educators from Central Europe (*M* = 3.43) and South Eastern Europe (*M* = 3.40) have much experience with interactive presentations. Educators from South Eastern Europe are also well experienced with online discussion boards/forums/wikis (*M* = 3.40). Striking values can be found in the following regions: Educators from Central Europe have no experience with digital educational games (*M* = 1.79). Educators from Northern Europe have no experience with VR/AR/XR (*M* = 1.43) and digital simulations (*M* = 1.43). Educators from Western Europe have no experience with OER (*M* = 1.77), digital simulations (*M* = 1.77), e-portfolios (*M* = 1.77), and VR/AR/XR (*M* = 1.62).

### Experience with Modalities and Tools Based on Professions

#### Modalities

##### Learners

**Figure 2:**
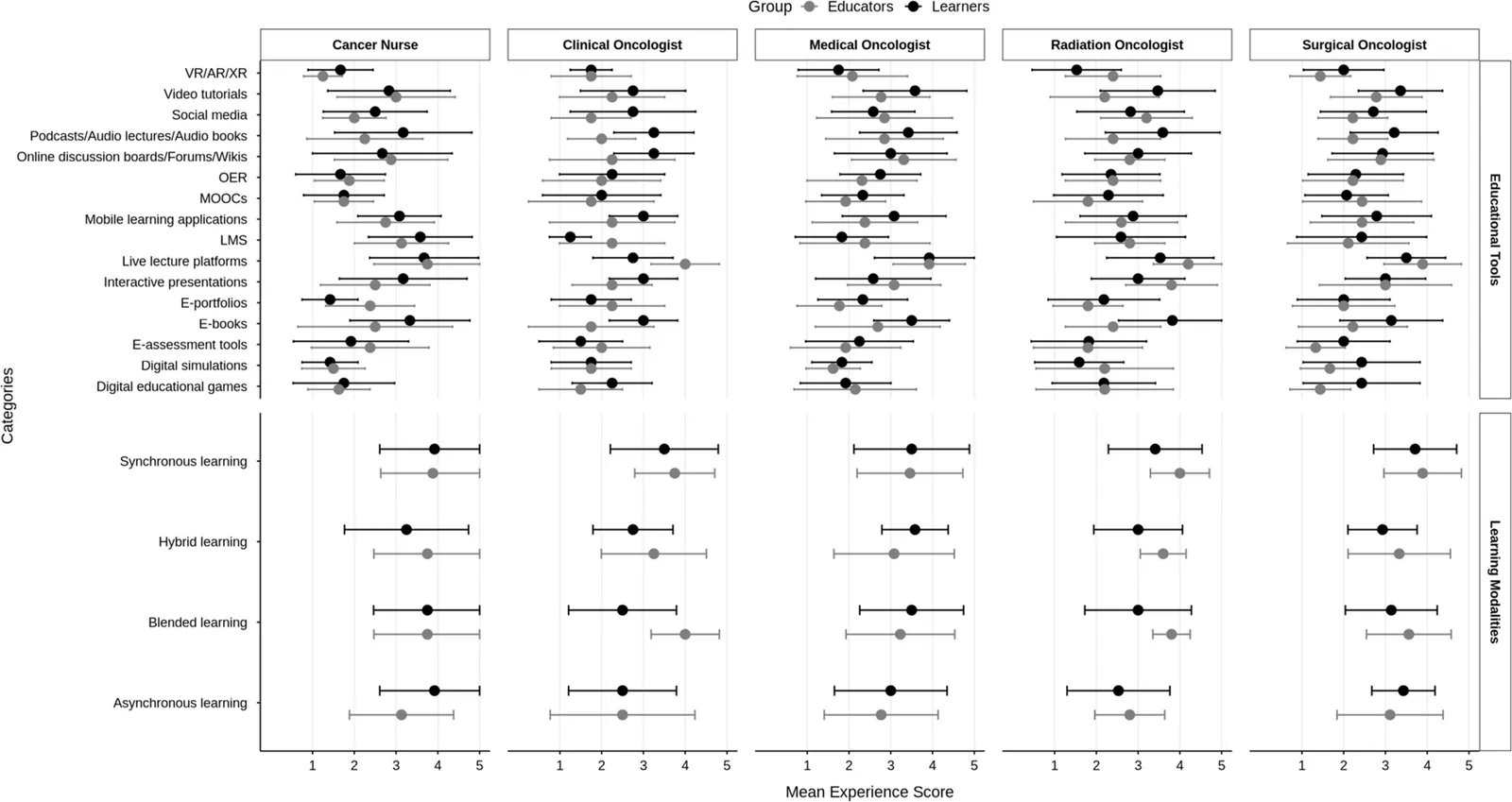
Experience with modalities and tools based on professions

Learners’ experience with modalities based on professions is presented in Fig. 2. Across professions, much experience can be found concerning synchronous learning. Whereas cancer nurses (*M* = 3.75), medical oncologists (*M* = 3.50), and pathologists (*M* = 3.56) also have much experience with blended learning, radiation oncologists (*M* = 3.00) and surgical oncologists (*M* = 3.14) reveal less experience with this modality. Learners within the field of clinical oncology demonstrate the least experience with blended learning (*M* = 2.50). As to asynchronous learning, cancer nurses are the most experienced (*M* = 3.92), clinical oncologists (*M* = 2.50), and radiation oncologists (*M* = 2.53) are the least experienced. Medical oncologists (*M* = 3.58) and pathologists (*M* = 3.50) have more experience with hybrid learning than the overall target group. Learners within the field of pathology have much experience with *all* modalities.

##### Educators

Compared to the overall sample, a similar pattern can be found across professions (Fig. 2), with pathologists indicating the highest value in synchronous learning (*M* = 4.33). As to blended learning, medical oncologists (*M* = 3.23) and pathologists (*M* = 3.00) have less experience than the overall target group. Among clinical oncologists, a rather low value can be found with regard to asynchronous learning (*M* = 2.50). Educators within the field of cancer nursing (*M* = 3.75) and radiation oncology (*M* = 3.60) are more experienced with hybrid learning compared to the overall sample.

#### Tools

##### Learners

Compared to the overall sample, a similar pattern can be found across professions (Fig. 2). Except for learners within the field of clinical oncology (*M* = 2.75), learners have much experience with live lecture platforms. Clinical oncologists are most experienced with podcasts/audio lectures/audiobooks (*M* = 3.25) and online discussion boards/forums/wikis (*M* = 3.25). Medical oncologists, radiation oncologists, and pathologists reveal much experience with video tutorials, podcasts/audio lectures/audiobooks, and e-books. Besides, cancer nurses have much experience with LMS (*M* = 3.58); pathologists have much experience with interactive presentations (*M* = 3.56) and social media (*M* = 3.69). Striking values can be found within the following professions: Learners within the field of cancer nursing have quite limited experience with MOOCs (*M* = 1.75), digital educational games (*M* = 1.75), OER (*M* = 1.67), VR/AR/XR (*M* = 1.67), digital simulations (*M* = 1.42), and e-portfolios (*M* = 1.42). Clinical oncologists have no experience with VR/AR/XR (*M* = 1.75), digital simulations (*M* = 1.75), e-portfolios (*M* = 1.75), e-assessment tools (*M* = 1.50), and LMS (*M* = 1.25). Learners within the field of medical oncology have no experience with VR/AR/XR (*M* = 1.75); radiation oncologists have no experience with digital simulations (*M* = 1.59) and VR/AR/XR (*M* = 1.53).

##### Educators

Educators’ experience with digital teaching tools based on professions is presented in Fig. 2. Across professions, educators are most experienced with live lecture platforms, with pathologists indicating the highest value (*M* = 4.33). Besides, radiation oncologists have much experience with interactive presentations (*M* = 3.80); pathologists demonstrate much experience with video tutorials (*M* = 3.67). Striking values can be found within the following professions: Again, educators within the field of cancer nursing have no experience with MOOCs (*M* = 1.75), digital educational games (*M* = 1.63), digital simulations (*M* = 1.50), and VR/AR/XR (*M* = 1.25). Clinical oncologists have no experience with MOOCs (*M* = 1.75), VR/AR/XR (*M* = 1.75), digital simulations (*M* = 1.75), e-books (*M* = 1.75), social media (*M* = 1.75), and digital educational games (*M* = 1.50). Educators within the field of medical oncology have no experience with e-portfolios (*M* = 1.77) and digital simulations (*M* = 1.62); surgical oncologists have no experience with digital simulations (*M* = 1.67), VR/AR/XR (*M* = 1.44), digital educational games (*M* = 1.44), and e-assessment tools (*M* = 1.33).

## Discussion

The findings from this exploratory study provide valuable insights into the experiences of cancer healthcare professionals with different digital learning modalities and tools across Europe. The results highlight varying levels of familiarity and engagement with synchronous, asynchronous, blended, and hybrid learning, as well as with specific digital tools such as live lecture platforms, video tutorials, e-books, and more advanced technologies such as VR/AR/XR and digital simulations. Comparisons were presented for both learners and educators to discuss whether certain regions or professions require special support during the implementation of the ISCTP.

Across Europe, both learners and educators revealed the highest level of experience with synchronous and blended learning modalities. The slightly higher experience ratings among educators compared to learners suggest that instructors are more comfortable facilitating synchronous online sessions, which might also be reflected in the increasing use of teleconference systems (e.g., Zoom and Microsoft Teams) during the COVID-19 pandemic. Findings related to blended learning indicate that many institutions have embraced a combination of face-to-face and online learning, facilitating self-paced online learning as well as offering individual learning paths [10]. In contrast, the comparatively low experience with asynchronous and hybrid learning indicates potential areas for growth [9]. The low levels of asynchronous learning acceptance, particularly among educators, might be connected to challenges in creating interactive and engaging content. In fact, the effectiveness of asynchronous learning is often limited by the lack of stimulation and participation [11]. Hybrid modes, although promising with regard to flexibility, might require greater institutional support and technological infrastructure to be fully realised in medical education.

When examining the tools used for digital education, live lecture platforms emerged as the most used tool by both learners and educators. This aligns with the preference for synchronous learning, in which immediate interaction and knowledge exchange are facilitated. In addition, learners also have substantial experience with e-books, video tutorials, and audio-based learning tools such as podcasts and audiobooks. These tools are flexible, allowing learners to perceive information at their own pace. The widespread use of these formats might be due to their accessibility and ability to complement busy professional schedules. Kulaksız et al. (2023) also reported that e-learning courses are the most used tool in digital oncology education, along with distance learning (including synchronous and asynchronous learning) being the most commonly used delivery mode. Despite positive attitudes towards different educational tools, advanced technologies such as VR/AR/XR, digital simulations, and e-assessment tools showed notably low experience ratings among the samples. These results also align with our previous research findings, indicating that TEL with advanced tools can be rarely found in oncology education [9]. Hindering factors regarding their use might include high costs, limited access to necessary hardware and software, and a lack of familiarity with these (mostly) innovative tools [12].

A closer look at the regional differences reveals important insights into how geographical factors might influence the adoption of different modalities and tools. While the overall patterns are similar across regions, notable deviations emerge. For instance, learners in Northern Europe reported lower experience with synchronous learning and live lecture platforms, but higher familiarity with podcasts and e-books. This could indicate a preference for mobile or distance learning formats. However, experiences with other tools that support autonomous learning (e.g., video tutorials, mobile learning applications, digital educational games) are limited. Central Europe showed higher levels of experience with asynchronous learning and interactive tools such as presentations, suggesting that this region has embraced more self-directed and collaborative digital education approaches. Nonetheless, experiences with e-assessment tools (including e-portfolios) remain limited. In contrast, Southern Europe consistently reported lower levels of experience with various modalities and tools, which might be reflective of regional disparities in digital infrastructure, accessibility, and institutional support.

The study also reveals differences between healthcare professions. Cancer nurses consistently reported the highest levels of experience with asynchronous learning (with the exception of the educator sample) and LMS, highlighting their ability for autonomous learning. This is also reflected in higher ratings of apps, video tutorials, and online discussion boards/forums/wikis among learners and educators compared to other educational tools. On the contrary, clinical oncologists demonstrated lower experience with various tools, including VR/AR/XR, digital simulations, and e-assessment tools. This might point to a gap in the availability or perceived relevance of these technologies for clinical oncology training, in which traditional methods might still be prioritised over digital alternatives. Similarly, radiation oncologists and surgical oncologists reported minimal experience with advanced tools such as VR/AR/XR and digital simulations, indicating that these technologies have yet to make inroads into these specialties, despite their potential for enhancing practical skill development. In general, it is important to recognise that internal (e.g., attitudes and beliefs, professional development opportunities) and external (e.g., institutional environment, workload, technology interactions) influences have an impact on the adoption and use of digital tools in instruction [13].

The results of this study lead to several implications for the development of TEL scenarios in the INTERACT-EUROPE project and future digital teaching/learning strategies in cancer care. The findings highlight varying levels of experience among cancer healthcare professionals with different digital education modalities and tools. Firstly, the complexity of the learning outcomes needs to be aligned with learners’ and educators’ previous experiences. Simple learning outcomes (e.g., understanding basic theoretical concepts or guidelines) might be more effectively addressed through widely used tools such as live lecture platforms, video tutorials, podcasts, and e-books. These tools are familiar to the majority of users, making them suitable for achieving less complex learning objectives with minimal additional support. Even though the degree of innovation appears to be rather low, we need to make sure that it can be accomplished by *all* participants and trainers of the ISCTP. Blind innovating might not necessarily lead to a successful and sustainable implementation of the programme. Complex learning outcomes (e.g., related to behavioural changes in professional practice) should be considered for on-site training in the cancer centres and should be complemented with (a)synchronous learning activities. Secondly, choosing the right modalities and tools for cross-regional as well as cross-professional oncology training involves balancing diverse target groups and implementation needs. Given the heterogeneity in the experiences of healthcare professionals across regions and professions, making a balanced decision about which modalities and digital tools to adopt is critical. While promoting multi-disciplinary learning, institutions should adopt a flexible approach that allows for customisation based on region, profession, and prior experiences of the target group. Providing alternative learning paths could ensure that a broader range of learners is accommodated. Targeted efforts are needed to bridge gaps in digital infrastructure, accessibility, and institutional support. Furthermore, economic factors and effectiveness can add another level of decision-making. While some of the tools signal the use of advanced technologies (e.g., AR/VR), their effectiveness might be limited to a very reduced set of learning objectives, while the costs of development are high compared to other solutions and scenarios.

The study had several limitations. First of all, the sample was a convenience sample, which was neither stratified by countries nor by professional background. This aspect limits the generalisability of the results, and in future survey studies, a stratified sample should be chosen. Furthermore, prior exposure to technology in undergraduate studies has not been assessed in this study. The nature of the study does not allow us to provide any explanations for the reasons for the identified differences. Future studies should use more complex variable constructs and theoretical models that can explain the differences.

## Conclusions

This study explored the previous experiences of cancer healthcare professionals with digital education modalities and tools across Europe. While certain modalities, such as synchronous and blended learning, and tools such as live lecture platforms are widely used and appreciated, advanced technologies remain underutilised. For innovations in oncology education to succeed, practitioners need to 1) align the complexity of learning outcomes with previous experiences and 2) balance diverse target groups and implementation needs, taking into account regional as well as professional disparities. Explanatory studies will be needed to confirm the preliminary findings.

## Supplementary Information

Below is the link to the electronic supplementary material.
ESM 1(PNG 44.2 KB)

## Data Availability

The data that support the findings of this study are openly available in OSF. Repository: Use of educational technologies in the oncology domain across Europe. https://doi.org/10.17605/OSF.IO/KD7GA This project contains the following underlying data: • learners_survey_387437_spss [transformed].sav • educators_survey_626933_spss [transformed].sav • Manuscript_Appendices_20241218.xlsx Data are available under the terms of the CC-By Attribution 4.0 International.
